# Audit of Timely Assessment, Diagnosis and Post-Diagnostic Support Provided by the West Leeds Memory Assessment Service

**DOI:** 10.1192/bjo.2022.478

**Published:** 2022-06-20

**Authors:** Daniel Romeu, Zumer Jawaid, Helen Turner

**Affiliations:** 1 Leeds and York Partnership NHS Foundation Trust, Leeds, United Kingdom; 2 Leeds Institute of Health Sciences, Leeds, United Kingdom

## Abstract

**Aims:**

In Leeds, Key Performance Indicators (KPIs) specify that patients should be offered an initial assessment within eight weeks of referral to the Memory Assessment Service (MAS) and diagnosed within 12 weeks. Additionally, post-diagnostic support (PDS) should be offered within two weeks of diagnosis. There are concerns that these targets are not being met due to the COVID-19 pandemic's impact on referrals and staff absence. This audit aims to establish whether the West Leeds MAS meets KPIs relating to the assessment and diagnosis of dementia and the provision of PDS in 80% cases.

**Methods:**

The 67 patients who were referred to the West Leeds MAS between 1 June and 31 July 2021 were included in this audit. Data were collected retrospectively from electronic patient records using an online proforma designed a priori. All data were quantitative and analysed descriptively using Microsoft Excel.

**Results:**

59 patients received an initial assessment; 19 (32%) received their initial assessment within 8 weeks, 14 (24%) had a delayed assessment with a documented reason, and the remaining 26 (44%) had a delayed assessment with no clear reason. 41 patients received a diagnosis; 23 (56%) received the diagnosis within 12 weeks, 12 (29%) had a delayed diagnosis with a documented reason, and 6 (15%) had a delayed diagnosis with no clear reason. Of those diagnosed, 25 (61%) were allocated a PDS appointment. No patients were offered PDS within 2 weeks of diagnosis, with no documented reasons for these delays.

**Conclusion:**

The MAS failed to meet the KPIs of interest, which may be partly explained by staffing issues and a backlog of referrals following the service's suspension in 2020. We aim to raise awareness of the KPIs, and the importance of documentation when KPIs cannot be met, by presenting at local meetings. We plan to liaise with clinical managers to identify systemic strategies to improve flow through the service while ensuring patient-centred care, and we will assess impact by repeating the audit in 12 months.

